# Quantum mechanical effects in plasmonic structures with subnanometre gaps

**DOI:** 10.1038/ncomms11495

**Published:** 2016-06-03

**Authors:** Wenqi Zhu, Ruben Esteban, Andrei G. Borisov, Jeremy J. Baumberg, Peter Nordlander, Henri J. Lezec, Javier Aizpurua, Kenneth B. Crozier

**Affiliations:** 1 Center for Nanoscale Science and Technology, National Institute of Standards and Technology, Gaithersburg, Maryland 20899, USA; 2 Maryland Nano-Center, University of Maryland, College Park, Maryland 20742, USA; 3 Material Physics Center CSIC-UPV/EHU and Donostia International Physics Center DIPC, Paseo Manuel de Lardizabal 5, Donostia-San Sebastián 20018, Spain; 4 Institut des Sciences Moléculaires d′Orsay - UMR 8214, CNRS-Université Paris Sud, Bâtiment 351, Orsay 91405, France; 5 Nanophotonics Centre, Cavendish Laboratory, University of Cambridge, Cambridge CB3 0HE, UK; 6 Department of Physics, MS61, Laboratory for Nanophotonics, Rice University, Houston, Texas 77005, USA; 7 School of Physics, University of Melbourne, Victoria 3010, Australia; 8 Department of Electrical and Electronic Engineering, University of Melbourne, Victoria 3010, Australia

## Abstract

Metallic structures with nanogap features have proven highly effective as building blocks for plasmonic systems, as they can provide a wide tuning range of operating frequencies and large near-field enhancements. Recent work has shown that quantum mechanical effects such as electron tunnelling and nonlocal screening become important as the gap distances approach the subnanometre length-scale. Such quantum effects challenge the classical picture of nanogap plasmons and have stimulated a number of theoretical and experimental studies. This review outlines the findings of many groups into quantum mechanical effects in nanogap plasmons, and discusses outstanding challenges and future directions.

## Abstract (web summary)

Recent work has shown that quantum mechanical effects in plasmonic nanogap structures become important as the gap distances approach the subnanometre length-scale. Here, the authors review the major findings which challenge the classical picture of these structures and discuss future directions for the field.

## Graphical abstract

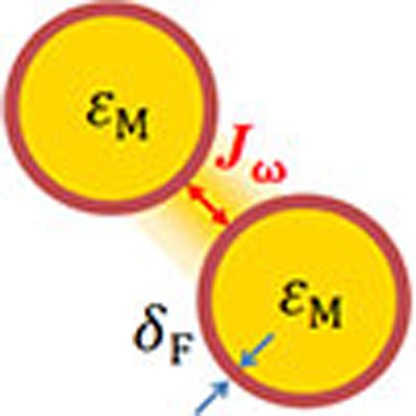

As a cornerstone of nanophotonics, the field of plasmonics, which studies the coupling between photons and collective oscillations of electrons, has seen tremendous development during the past few decades^1^,^2^. Originally, research in this field was mainly concerned with the resonant interaction between light and metallic nanostructures that enables localization of electromagnetic fields at the sub-wavelength scale, as well as enhancement of optical absorption and scattering phenomena^3^,^4^. The field has quickly expanded to an interdisciplinary one that spans optics^5^,^6^, material science^7^,^8^, chemistry^9^, biology^10^ and energy^11^, owing to the flexibility with which the plasmonic resonances can be engineered^12^. Interest in metallic structures with nanogap features has been propelled mostly by important applications in spectroscopy^13^,^14^,^15^, and in particular, by the experimental reports of surface-enhanced Raman scattering (SERS) from single molecules^16^,^17^,^18^. The extreme level of sensitivity possible with these structures arises from the localized surface plasmon resonances associated with the nanogap^3^,^19^, in which electric field intensity enhancements can reach ≈10^4^. The general practice in modelling such plasmonic resonances is based on classical electromagnetic theory, where the collective motion of the electrons is lumped into empirical or model local dielectric constants of the materials^1^ and the distribution of the electromagnetic field is given by the solution of Maxwell's equations. This approach predicts monotonically increasing electric-field enhancements with decreasing gap distance, prompting the development of nanotechnology for producing plasmonic structures with ever-smaller gaps^20^,^21^,^22^,^23^,^24^,^25^,^26^,^27^.

Remarkably, recent theoretical^28^,^29^,^30^,^31^ and experimental^32^,^33^ advances show that as the gap distance enters the nanometre and then subnanometre scale, the quantum nature of the electrons and the nonlocal screening^34^,^35^ associated with them significantly alter the plasmonic response. In this quantum regime, the classical descriptions fail to account for the actual localization of the surface charges induced by an incident electromagnetic field^36^,^37^,^38^,^39^,^40^. The spatial localization of the surface charges can be characterized by the frequency-dependent distance parameter *δ*_F_, the Feibelman parameter^41^, which is in the angstrom (Å) range. *δ*_F_ is defined as the position of the centroid of the induced surface charge density with respect to the geometrical boundaries^36^,^37^,^38^,^39^,^40^. The shift of the induced surface charges with respect to the geometrical boundaries of the metal is intimately related to the nonlocal screening of the electrons and leads to an ‘effective' modification of the metal interface boundaries, creating an ‘effective' gap distance that also differs from the geometrical value^42^,^43^. This effect can be described using model nonlocal dielectric functions with different levels of sophistication in the theoretical descriptions^44^,^45^,^46^,^47^,^48^,^49^,^50^,^51^.

Electron tunnelling across the gap at optical frequencies is another relevant quantum feature that cannot be captured by a classical theory. The process of electron tunnelling through a potential barrier at lower frequencies is extensively discussed in quantum mechanics. Examples of this effect include the strong field ionization of atomic species that is used in the generation of attosecond electromagnetic pulses^52^ and scanning tunnelling microscopy (STM)—one of the most powerful methods of surface analysis^53^,^54^. The tunnelling current in an STM junction is triggered by the applied direct-current (*dc*) bias between the tip and the surface. In the alternating-current (*ac*) regime, photon-assisted tunnelling for metal-dielectric-metal or semiconductor junctions has been thoroughly studied in the terahertz range^55^,^56^,^57^,^58^. It is only recently that quantum mechanical theories^59^,^60^,^61^,^62^,^63^,^64^,^65^,^66^ and experimental studies performed in well-controlled metallic gap junctions^67^,^68^,^69^,^70^,^71^,^72^,^73^ have started to address the tunnelling phenomena in plasmonics at optical frequencies. For a gap distance comparable with the length-scale of the electron spill-out from the interfaces, the electron densities at the metal surfaces start to overlap. This means that the conduction electrons can tunnel through the potential barrier across the junction at optical frequencies, a precursor of the formation of touching metal surfaces. The existence of such a phenomenon is essential to explain the observed smooth transition of the plasmonic response upon variation of the geometry from a subnanometre gap to touching metal surfaces^74^,^75^,^76^.

We schematically illustrate the influence of these quantum mechanical effects on plasmonic resonances of nanogap structures in Fig. 1. This figure depicts the energies of the plasmonic modes as the gap distance between a pair of particles in vacuum is decreased until they merge. Large gap distances correspond to the classical regime, for which the local Maxwell's equations correctly describe the red-shift of the gap plasmon modes. As the gap distance becomes smaller than a few nanometres (that is, below ≈10*δ*_F_), the system enters the quantum regime, requiring a more detailed treatment. In the distance range where the conductance between the particles remains sufficiently small, the nonlocal screening is the dominant quantum effect on the optical response. The evolution of the plasmon modes is qualitatively similar to that predicted by the local classical model, but quantitative differences emerge. To correctly quantify the nonlocal screening effects and associated changes in plasmonic response, the surface charge distribution must be considered accurately. As the gap distance continues to decrease and the geometry is characterized by gaps narrower than a ‘threshold tunnel-distance' *d*_th_, the electron tunnelling effect completely modifies the behaviour of the plasmonic response: the red-shifting gap plasmon modes progressively disappear and the blue-shifting charge-transfer plasmons (CTPs) gradually emerge. Most importantly, this transition occurs before the two particle surfaces touch. The distance *d*_th_ separating the nonlocal and tunnelling regimes is typically in the subnanometre range. It can be understood as the separation at which the tunnelling-induced conductance becomes large enough to allow a significant fraction of the plasmon-induced surface charges to tunnel across the gap in half an optical cycle. Such threshold conductance can be estimated by *σ*_th_≈*cɛ*_0_/*λ*, where *c* is the speed of light, *ɛ*_0_ is the vacuum permittivity and *λ* is the wavelength^67^,^76^.

In this review, we first outline theoretical advances regarding quantum mechanical effects in plasmonic structures with subnanometre gaps. We then describe experimental studies that have revealed the presence of these quantum effects on plasmonic resonances. We further discuss potential applications of combining quantum effects in nanogaps with plasmonic excitation. These exciting studies and their on-going development clearly show the vitality of this research topic, which opens new regimes in nanophotonics, bridging plasmonics, and electron transport in nanoscale gap junctions.

## Modelling quantum mechanical effects in the plasmonic response

**Figure 2:**
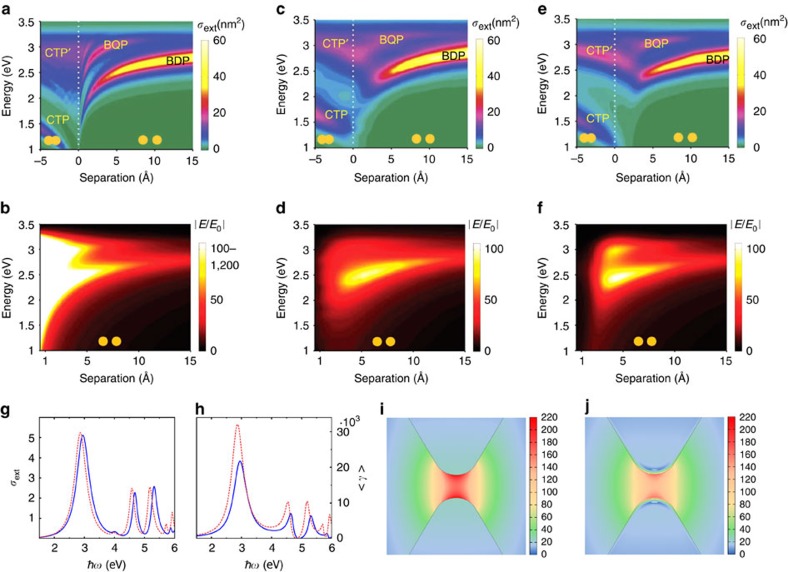
Modelling of plasmonic dimers with subnanometre gap. (**a**–**f**) Comparison of the optical properties of a metallic dimer simulated with (**a**,**b**) local Drude model, (**c**,**d**) TDDFT calculation and (**e**,**f**) the QCM. The dimer consists of two Na spheres with radii of 2.17 nm. (**a**,**c**,**e**) The colour plots of the extinction spectra *σ*_ext_ as a function of gap distances. (**b**,**d**,**f**) The simulated near-field enhancements as a function of gap distances. The relevant plasmon modes are labelled: bonding dimer plasmon (BDP), bonding quadrupolar plasmon (BQP), charge transfer plasmon (CTP) and higher-order charge transfer plasmon (CTP'). (**a**–**f**) Images are reproduced from ref. 64. Copyright 2014 Nature Publishing Group. (**g**–**j**) Simulations of nonlocal effects for a metallic bowtie dimer. The dimer consists of two Au wires with cross-sections comprising equilateral triangles (side length: 45 nm, gap distance: 1 nm, tip radius of curvature: 1 nm). (**g**) Extinction cross-section spectra *σ*_ext_ and (**h**) the near-field enhancement <*γ*> calculated from the local classical model (red dashed lines) and the NLHD model (blue solid lines). (**i**,**j**) Near-field distribution around the gap region of the Au bow-tie dimer calculated from (**i**) the local classical model and (**j**) the NLHD model. The colour scales in **i**,**j** represent the enhancement of the electric near-field. (**g**–**j**) Figures adapted with permission from ref. 86.

We start by discussing in detail the classical electromagnetics description of plasmons in a nanoparticle dimer, that is, a pair of nanoparticles separated by a small gap^21^. In classical modelling of the plasmonic interaction between light and free electrons, Maxwell's equations are employed to describe the oscillating electromagnetic field, and the collective motion of the electrons is lumped into the metal permittivity. In the ‘local response' approximation, the metal permittivity is taken as a local function given by empirical data^77^ or by model descriptions, for instance the Drude model^1^. This approximation has proven effective for simulations of many plasmonic nanostructures^3^,^4^,^5^,^6^, and we refer to such electrodynamic modelling as the ‘local classical model'. Figure 2a shows local classical simulations of the extinction cross-section spectra of a small dimer of sodium (Na) spheres (described through the Drude model) as a function of gap distance *d*. Na is often used for such calculations because it is a good prototype for a free electron metal, and thus appropriate as a reference for comparison with approaches that incorporate quantum mechanical effects^64^. For finite gap distances, the fundamental gap plasmon mode of the dimer is the bonding dimer plasmon (BDP), in which the plasmons in the two nanospheres are ‘bonded' through Coulomb interaction between charges of opposite signs distributed across the nanogap region^78^,^79^. The local classical model predicts that the peak frequency of the BDP monotonically red-shifts as the gap distance decreases. In addition, it predicts dramatic enhancement of the electric field in the gap centre for small gap distances (Fig. 2b). In the regime where the nanospheres overlap (negative gap distances in Fig. 2a), CTP modes^74^,^75^,^76^ appear and blue-shift as the nanospheres merge together.

A close look at Fig. 2a,b suggests that the local classical model exhibits unphysical behaviour as the gap distance tends to zero. First, in this regime, slight changes in the gap distance result in extraordinarily large frequency-shifts of the BDP. Similar divergent frequency-shifts of the CTP modes are also predicted for slightly overlapping nanospheres. As we discuss in later sections, experimental observations show that these do not occur. Second, an abrupt change of the plasmonic modes is predicted when the geometry changes from separated to overlapping dimer^75^. In addition, the local classical model predicts diverging field enhancements for vanishing gaps^3^,^19^. These unphysical behaviours in the subnanometre regime stimulated a more rigorous investigation of how quantum mechanical effects influence plasmon resonances.

In a pioneering study, Zuloaga *et al*.^59^ used time-dependent density-function-theory (TDDFT) to perform fully quantum-mechanical simulations of the linear response of plasmonic dimers. Other groups extended this study to treat both linear and nonlinear effects in a variety of gap configurations^31^,^42^,^61^,^62^,^63^. Most of these simulations employ a jellium model for the plasmonic material to describe the temporal evolution of the electron densities and currents induced in the system. In the jellium model, the ionic cores of the atoms are represented by a uniform background charge density, with the jellium edge located at a distance of half the lattice constant from the last atomic plane at the surface^59^. Because of its simplicity, the jellium model enables the fully quantum mechanical description of metallic nanoparticles that are large enough to support plasmon resonances^34^. Figure 2c,d shows the TDDFT-simulated extinction cross-sections and field enhancements of the dimer considered in Fig. 2a,b, under sufficiently weak illumination to avoid nonlinear effects. These results clearly demonstrate the existence of quantum effects for plasmonic structures with subnanometre gaps: (i) the red-shift of the bonding modes is smaller than that predicted by the local classical simulation; (ii) the BDP and higher-order bonding modes progressively disappear before the gap distance reaches zero; (iii) the CTP modes of the dimer emerge prior to the direct geometrical overlap of the two Na spheres; (iv) along with the extinction of the bonding modes, the electric near-field enhancement is quenched before the two Na spheres are in geometrical contact. Thus, in contrast to the local classical calculations, the quantum results point towards a continuous transition from a capacitive to a conductive coupling across the gap^76^.

More sophisticated quantum treatments are possible in which the nanoparticles are described atomistically^80^,^81^,^82^. Results at the full atomistic level show the sensitivities of the far- and near-field optical responses to the exact morphology of the plasmonic gap, and to the presence of atomic-scale features such as crystallographic edges and vertices at the boundaries between atomic planes forming the nanoparticle surface. Notably, the lightning rod effect at the atomistic level might allow for subnanometre confinement, with important implication for spectroscopies and microscopies^83^. Despite these quantitative details, it is important to stress that the overall behaviour of the resonances is captured well by the jellium model.

It is computationally unfeasible, however, to perform TDDFT simulations for large plasmonic structures (sizes⪞10 nm) that are commonly used in plasmonics, because they contain too many electrons to allow first-principles modelling^60^. To address this situation, semi-classical approaches have been proposed (Box 1) to incorporate the quantum effects into the framework of Maxwell's equations. In the following, we first concentrate on the semi-classical modelling of the electron tunnelling effect. As is well established in STM^53^, the spill-out of the electron density outside the surface leads to electron tunnelling across the gap. The results for plasmonic dimers in the quantum regime can be explained by considering the electron tunnelling at optical frequencies: the onset of the electron tunnelling provides an effective ‘charge transfer' channel, neutralizing the bonding-plasmon-induced charges of opposite signs across the gap and thus quenching the field enhancement^59^,^60^. Moreover, such electron tunnelling implies a resistance^55^,^56^,^57^,^58^ that broadens the plasmon resonances. As the bonding modes vanish, the CTPs are established because the two nanoparticles forming the plasmonic dimer are conductively connected through electron tunnelling as if they were bridged physically. Hence, for large plasmonic structures, Esteban *et al*.^64^,^65^ developed a quantum-corrected model (QCM) to incorporate such electron tunnelling effect into the local classical formalism. The quantum relationship between the oscillating field and current is reproduced by assigning to the gap a local effective conductivity *σ*_*g*_(*ω*) (see Box 1 for the technical description). It can be seen that the QCM calculations (Fig. 2e,f) are in good agreement with the results of TDDFT (Fig. 2c,d). By comparing these results, it is found that the ‘threshold tunnelling-distance' *d*_th_ is ≈4 Å in this case. For larger gap distances, the QCM results are identical to those from the local classical model (Fig. 2a,b) as the tunnelling effect is negligible. Following this work, refined treatments have been proposed^84^,^85^ based on the theory of laser-assisted tunnelling in metal–dielectric–metal junctions^55^,^56^,^57^,^58^. In another effort, Hohenester *et al*.^66^ implemented the QCM in a boundary-element-method approach by modifying the boundary conditions of the plasmonic structures. The results showed good agreement with the original QCM developed by Esteban *et al*.

Along with electron tunnelling, nonlocal screening^34^,^35^ is another quantum effect that could influence the plasmon resonances of a metallic dimer. It refers to the fact that, because of electron–electron interactions, the motion of the conduction electrons at each point in space depends not only on the field applied at that point but also on fields at other points^28^,^29^,^30^. The nonlocal screening prevents sharp charge localization at interfaces and is therefore important for small particles^36^,^37^,^38^,^39^ and narrow gaps^28^,^29^,^30^,^31^,^32^. Among different methods developed to address nonlocal effects^46^,^47^,^48^,^49^, the nonlocal hydrodynamic (NLHD) model^44^,^45^ has been widely used in the context of plasmonics because of its physical transparency, as well as analytical and numerical efficiency. In this approach (Box 1), the collective motion of electrons is governed by the linearized hydrodynamic Navier–Stokes equation. Unlike the local classical model in which the plasmon-induced charge densities are strictly confined to the surfaces, in the NLHD description these charges are effectively pushed inside the material. This is because of the choice of the boundary conditions on the free electron current at the metal–vacuum interface^29^,^30^. As a result, the plasmon resonances obtained with the NLHD approach are always blue-shifted with respect to the wavelengths predicted by the local classical model (Fig. 2g)^86^. The nonlocal screening effect also reduces the electric field enhancement in the gap for plasmonic dimers (Fig. 2h–j). Indeed, these behaviours predicted by NLHD model correctly mimic the measured effect of nonlocal screening for noble metals such as Au^32^ and Ag^87^.

Interestingly, the NLHD model fails to describe the nanoparticle size effects on plasmon energies in free-electron materials such as alkali metals or aluminum, with its predicted blue-shifts being contrary to the red-shifts that are measured^88^ and obtained using the TDDFT calculations^89^. These plasmon energy shifts are ultimately related to the microscopic spatial distribution of the plasmon-induced screening charges^42^,^43^. The screening charges are pushed into the material in noble metals (Au and Ag, where delocalized *s*-*p* and localized *d*-electrons participate in the screening of external fields), but are slightly displaced out of the metal surface in alkali metals and aluminum (where only conduction *s*-*p* band electrons provide the screening). As pointed out by Teperik *et al*.^42^, if one accounts for the actual position of the plasmon-induced charges, local classical calculations with an ‘effective' gap distance can be used to recover the nonlocal effects. Similarly, Toscano *et al*.^43^ also showed that the NLHD model could be greatly improved by accounting for the electron density profile at the surface. This crucial input, together with the consideration of a polarizable background accounting for *d*-electron screening, allows consistent results to be obtained for nanoparticles of different metals.

Theoretical efforts have also been made in developing generalized models that cover both the tunnelling and the nonlocal regimes. It has been shown that the QCM approach can be implemented in the NLHD model (NLHD-QCM)^65^,^90^, allowing simultaneous treatment of both nonlocal screening and tunnelling effects. Meanwhile, Mortensen *et al*.^51^ extended the hydrodynamic approach and proposed a generalized nonlocal response model in which an electron diffusion term is added to the NLHD model, resulting in a progressive broadening of bonding modes in the subnanometre gap regime that is in line with quantum TDDFT results. However, contrary to TDDFT calculations, the generalized nonlocal response model simulations do not show the establishment of CTPs in the gap distance regime of 0≤*d*≤*d*_th_. This difference indicates that the electron tunnelling effect needs to be accounted for in order to fully understand the optics of subnanometre gaps.

In addition to the modification of far- and near-fields in the linear plasmonic response, quantum effects can lead to a variety of nonlinear effects. For example, Marinica *et al*.^62^ and Wu *et al*.^84^ showed that strongly enhanced fields could trigger tunnelling currents at gap distances for which there would be negligible tunnelling in the linear response regime. This electronic discharge could produce high-frequency components of the electron current across the gap, and offers interesting prospects for attosecond pulse generation^52^. These new phenomena and the on-going theoretical developments clearly show the vigour of this research topic.

## Realization of metallic structures with subnanometre gaps

Much of the interest in developing theoretical models to describe quantum mechanical effects for plasmons in nanogap structures was prompted by the emergence of reliable fabrication methods for such structures. Previous methods for fabricating narrow gap structures typically relied on top–down electron-beam lithography (EBL)^4^,^5^,^6^ approaches or bottom–up approaches based on the self-assembly of nanoparticles^21^. The resolution of single-exposure EBL is primarily limited by electron scattering in the substrate, limiting gap distances down to ≈2 nm with fair yield^26^, while sometimes^24^ enabling gap distances of ≈5 Å. In bottom–up methods, the gap distances achieved are typically defined by the sizes of the surfactant molecules used to assemble the plasmonic nanoparticles. Previous efforts had focused on the use of relatively large molecules such as cetyltrimethyl ammonium bromide^91^ or DNA^22^ resulting in gap distances around ≈1 nm. In this section, we summarize some recent key fabrication approaches that have enabled the experimental observation of tunnelling and nonlocal effects. These works can be generally categorized into the ‘tip-based' and ‘nanoparticle-based' gap-configurations.

**Figure 3:**
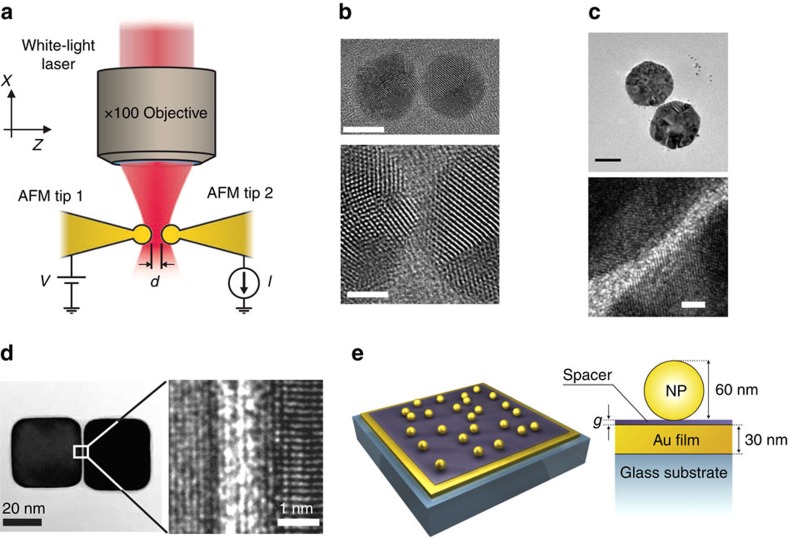
Subnanometre gaps formed in metallic structures. (**a**) Schematic illustration of the plasmonic dimer formed by pushing two AFM cantilevers in tip-to-tip configuration. Reproduced from ref. 67. Copyright 2012 Nature Publishing Group. (**b**) High-resolution TEM images of Ag nanosphere dimer formed by pushing two Ag nanoparticles together using focused electron beam. The diameter of each Ag nanoparticle is 9 nm. Top panel: overview of the dimer. Scale bar is 5 nm. Bottom panel: Zoomed-in view of the Ag dimer showing the gap distance *d*≈3.5 Å. Scale bar is 2 nm. Reprinted with permission from ref. 68. Copyright 2013 American Chemical Society. (**c**) TEM image of Au nanodisk dimer form by two-step electron beam lithography. The diameter of each nanodisk is 90 nm. Top panel: overview of the dimer. Scale bar is 50 nm. Bottom panel: zoomed-in view of the Au dimer showing the gap distance *d*≈2.0 Å. Scale bar is 2 nm. Reproduced from ref. 70. Copyright 2014 Nature Publishing Group. (**d**) High-resolution TEM image of the Ag dimer formed by linking molecules, with zoomed-in TEM image showing the subnanometre gap distance. Image reproduced from ref. 97. Copyright 2014 AAAS. (**e**) Schematic of particle-on-film geometry, with the nanoscale gap defined by a thin self-assembled monolayer layer. Image reproduced from ref. 32. Copyright 2012 AAAS.

‘Tip-based' gap-configurations are usually implemented by modifying atomic force microscopes (AFMs) or STMs. These configurations enable precise gap control^53^,^92^ in the subnanometre regime. They are advantageous in that the metallic tips and/or surfaces act as electrodes, enabling the estimation of the gap distance through conductivity or tunnelling-current measurements^54^,^57^ and simultaneous optical characterization of the plasmonic resonances. For example, Savage *et al*.^67^ showed that gap distances in the tunnelling regime can be achieved by pushing two gold-coated AFM cantilever tips together using a piezoelectric stage (Fig. 3a). Subnanometre gap distances can be achieved, with physical contact between the tips indicated when the *dc* tunnelling conductance *G* across the gap exceeds the quantum conductance *G*_0_=2*e*^2^/*h* (where *e* is the elementary charge and *h* is the Planck's constant). The ability to sweep across a wide range of the gap distances using this method is helpful in identifying and studying quantum effects. Using similar configurations, sharp tips have been brought to subnanometre distances from planar surfaces by monitoring the *dc* tunnelling current^53^ or the shear force^71^,^72^. Tip-based structures with subnanometre gaps have also been produced by electromigration^93^.

The ‘nanoparticle-based' configuration with subnanometre gaps offers the advantage of allowing direct imaging of the gap junction, for example, with a transmission electron microscope (TEM) capable of providing atomic-scale resolution. Such ‘nanoparticle-based' gap structures have now been demonstrated using innovative fabrication methods. One novel bottom–up approach for forming such structure was recently demonstrated by Scholl *et al*.^68^,^69^, who used electron beams to manipulate plasmonic nanoparticles of sizes up to ≈25 nm (Fig. 3b). In this method, a focused electron beam induces an attractive Coulomb force between two closely spaced Ag nanoparticles, causing them to move even closer and subsequently merge. The nature of the stress that moves nanoparticles together involves the presence of electromagnetic forces induced by the plasmonic fields, together with other less straightforward effects such as particle diffusion. These effects overcome the cohesion forces between the particles and substrate, thus producing a latched motion that merges the particles^94^. The nanoparticle velocity was controlled by the intensity of the electron beam and can be as low as 0.2 Å s^−1^, making it possible to image the nanoparticles via TEM while simultaneously monitoring the plasmon modes via electron energy loss spectroscopy^95^.

Subnanometre gap distances between two larger (sizes ⪞25 nm) nanoparticles have also been achieved using a top–down process that involves the use of two EBL steps^70^. The two parts of the dimer are defined sequentially in separate EBL steps, thereby circumventing many of the resolution challenges facing EBL patterning. This method allows the fabrication of dimers with gap distances down to ≈2 Å (Fig. 3c). It also yields dimers fixed to a TEM membrane, meaning that the results of the optical and structural characterization of each dimer can be compared, although the mobility of Au atoms at room temperature means great care has to be exercised in their treatment.

Bottom–up approaches based on small surfactant molecules^23^,^96^,^97^ or inorganic two-dimensional van der Waals spacer materials^27^ present other opportunities for achieving subnanometre gaps in large plasmonic dimers. Organic molecules with thiol-groups are typically used because of their strong bonds to Au/Ag surfaces and their appropriate sizes (≈3 Å to ≈2 nm) for this application. Tan *et al*.^97^ reported that dimers with gap distances of ≈4 Å could be obtained in this way with yields of ≈30% (Fig. 3d). Small surfactant molecules have also been used as dielectric spacers in the particle-on-film geometry (Fig. 3e)^32^,^33^. In these configurations, the gap distance created by the molecular spacer were either measured via ellipsometry (for thicknesses >2 nm) or estimated based on the number of chain-linkers of the molecules (for thicknesses <2 nm). Recent work has also demonstrated the possibility of narrowing the gap to the subnanometre level by illuminating the spacer molecules with an external illumination source that induces photo-oxidative desorption^98^.

Several other techniques have also been developed to achieve subnanometre gaps in plasmonic structures^99^,^100^,^101^. As we discuss in the following sections, these novel techniques provide important insight into quantum effects in plasmonics. As an active research area in itself, the development of nanogap fabrication methods will continue to propel the field of plasmonics.

## Observations of quantum effects via far-field measurements

Recent success in fabricating plasmonic structures with subnanometre gaps enable the experimental examination of the quantum mechanical effects in these structures. As discussed in the theory section, the quantum nature of the nanogap plasmons leads to four major linear effects that cannot be captured by local classical theories: (i) a non-divergent frequency shift of plasmonic modes; (ii) the progressive vanishing of the bonding plasmon modes in narrow gaps; (iii) the emergence of charge-transfer plasmon modes before direct physical contact; (iv) the quenching of field enhancement. In this section, we highlight several key optical far-field studies (related to effects (i)–(iii)) that have demonstrated the impacts of the quantum mechanical effects on plasmonic resonances. These studies also provide insight into the validity of the semi-classical approaches for modelling quantum effects in plasmonics. The discussion of the near-field effect (iv) is addressed in the next section.

**Figure 4:**
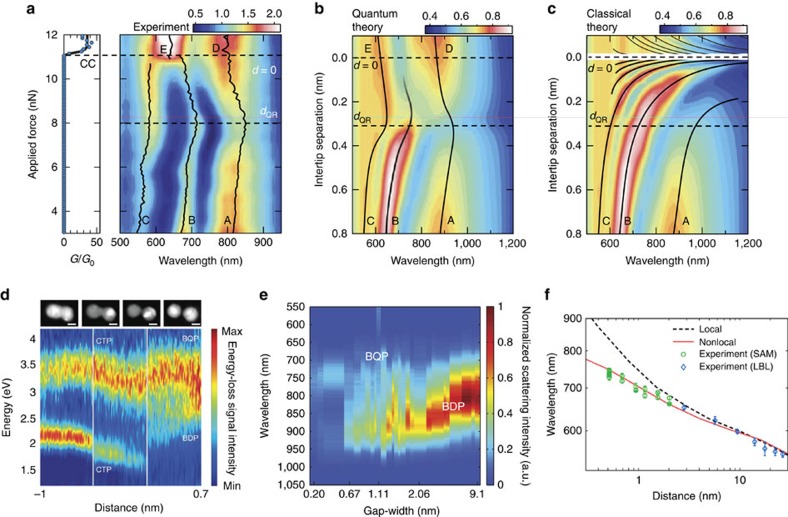
Experimental observations of quantum mechanical effects in plasmonic dimers. (**a**) Simultaneously measured electrical conductance (*G*/*G*_0_) and dark-field scattering of the AFM dimer shown in Fig. 3a with increasing force applied to the inter-tip cavity after snap-to-contact. The bonding plasmon modes are labelled as A, B and C. The CTP modes are labelled as D and E. (**b**) QCM and (**c**) local classical model simulations of the tip-based dimer. The colour scales in **a**–**c** represent relative scattering intensities. (**a**–**c**) Reproduced from ref. 67. Copyright 2012 Nature Publishing Group. (**d**) EELS spectra of the Ag dimers shown in Fig. 3b as the gap distance changes from 7 Å to –1 nm. STEM images (top panels) collected at the beginning and end of each scan (highlighted by white solid vertical lines) indicate the gap distances of +7 Å,<2.7 Å, −3 Å and −1 nm from right to left. Reprinted with permission from ref. 68. Copyright 2013 American Chemical Society. (**e**) Dark-field scattering spectra of the EBL-fabricated Au nanodisk dimers shown in Fig. 3c with various gap distances. Reproduced from ref. 70. Copyright 2014 Nature Publishing Group. (**f**) Peak wavelengths (green circles and blue diamonds) of the dark-field scattering spectra of the particle-on-film structure shown in Fig. 3e as a function of gap distance. Wavelengths deviate from expectations of local classical simulations (black dashed line) at small gap distances, but can be explained with nonlocal models (solid red line). Error bars represent the uncertainty in determining the wavelength of the plasmon resonance peak. Image reproduced from ref. 32. Copyright 2012 AAAS.

The tunnelling effect and the QCM model were first examined by Savage *et al*.^67^ using the tip-based gap-configuration depicted in Fig. 3a. The dark-field scattering spectra recorded as the gap distance *d* reduces from the nanometre scale to the touching state (*G*≥*G*_0_) are shown in Fig. 4a. Also included are the predictions from the QCM approach (Fig. 4b) and the local classical model (Fig. 4c). Two regimes can be identified from the measured spectra, demarcated by the gap distance *d*_QR_ that denotes the onset of the quantum regime (note that *d*_QR_ is the same as the threshold tunnel-distance *d*_th_ defined in the Theory section). For *d*≥*d*_QR_, the peak positions of all three bonding plasmon modes (labelled as A, B and C) red-shift as gap distance *d* decreases, a behaviour that is consistent with the local classical models. For *d*≤*d*_QR_, effects (i)–(iii) are indeed observed: the measured bonding modes gradually evolve into the CTP modes (labelled as D and E), with a blue-shift of the peak positions as *d* is further reduced. These effects clearly support the existence of the tunnelling effect predicted by the QCM model (Fig. 4b), while significantly deviating from the predictions of the local classical model (Fig. 4c). This behaviour is also generally consistent with the predictions of TDDFT calculations for much smaller structures described in the Theory section.

The impact of the tunnelling effects on plasmonic resonances have also been observed for nanoparticle-based dimers. Scholl *et al*.^68^ performed correlated TEM and electron energy loss spectroscopy measurements on a dimer consisting of two small Ag nanoparticles with diameters of ≈9 nm (Fig. 3b). The results (Fig. 4d) are consistent with the general behaviour predicted by the TDDFT^62^ and QCM^64^,^65^, revealing a ‘threshold tunnel distance' of around 5 Å. This work has recently been extended to trimers^69^. Meanwhile, Zhu *et al*.^70^ used TEM to image plasmonic dimers comprising two large Au nanodisks (Fig. 3c). The plasmon resonance of each dimer was characterized by dark-field scattering spectroscopy (Fig. 4e). The tunnelling regime was reached for gap distances below 7 Å, as indicated by the suppression of the bonding plasmon modes. These results, together with the studies on tip-based structures, provide strong evidence for electron tunnelling in plasmonic structures with sub-nanometre gaps.

To examine the nonlocal screening effect in nanoscale gaps, Ciracì *et al*.^32^ studied the plasmon resonances of the particle-on-film structure, with the gap formed via molecular spacers (Fig. 3e). The peak position of the plasmonic resonance was found to monotonically red-shift as the gap distance reduced from ≈11 nm to ≈5 Å (Fig. 4f) though accurately measuring these separations is not without problems (as for many of the techniques here). This red-shifting trend is qualitatively consistent with the local classical model^75^,^78^. However, for gap distances less than ≈3 nm, the measured peak wavelengths deviate considerably from the predictions of the local classical model. These deviations can instead be accounted for by the NLHD, highlighting the importance of the nonlocal effects. Zhu *et al*.^70^ also found that the measured peak positions for plasmonic nanodisks with subnanometre gaps were generally in agreement with QCM simulations, but with some differences that could be due to other effects such as the nonlocality and the morphology of the nanoparticles and the gap^80^,^81^,^82^,^102^,^103^.

## Quenching of plasmonic near-field enhancement

One of the main motivations for fabricating plasmonic substrates with nanoscale gaps has been the optimization of plasmonic enhancement, that is, the generation of intense electric fields confined to subwavelength dimensions^21^. As discussed in the Theory section, one can expect plasmonic field enhancement to be reduced by the onset of tunnelling and nonlocal screening (effect (iv)). In this section, we highlight experimental studies of both linear and nonlinear optical processes in subnanometre gap structures that have demonstrated quenching of plasmonic enhancement by quantum mechanical effects.

**Figure 5:**
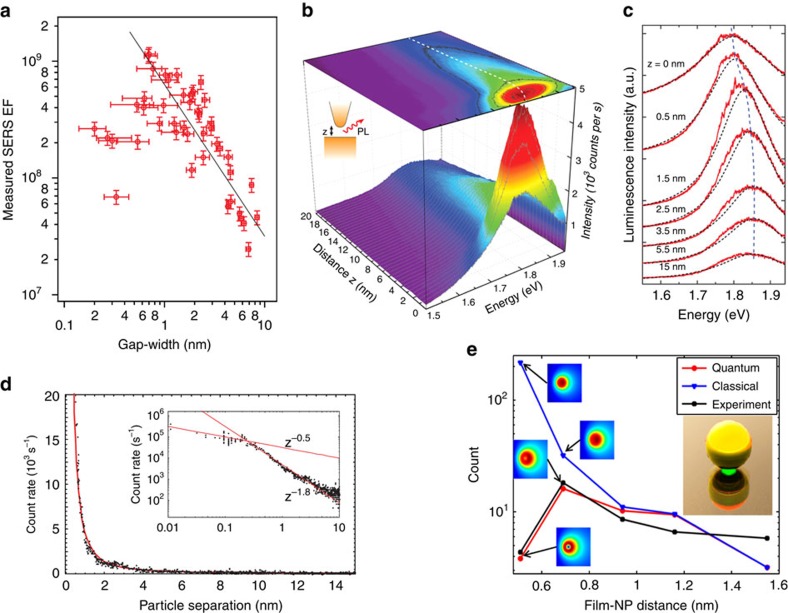
Quenching of plasmonic enhancement at subnanometre gaps. (**a**) SERS enhancement factors measured for Au dimers shown in Fig. 3c with various gap distances. Largest enhancement is obtained for dimer with gap distance ≈6.7 Å. The horizontal error bars represent the uncertainties in retrieving the gap-width from TEM images. The vertical error bars originate from the uncertainties in the reference Raman measurements. Reproduced from ref. 70. Copyright 2014 Nature Publishing Group. (**b**) Gap distance dependence of photoluminescence intensity in STM-based measurements (experimental configuration schematically illustrated in inset). (**c**) Corresponding PL spectra for different gap distances in these STM-based measurements. (**b**,**c**) Images are reprinted with permission from ref. 72. Copyright 2014 American Chemical Society. (**d**) Four-wave mixing photon count rate as a function of gap between two 60-nm-diameter gold nanoparticles. The inset shows a detailed view on a log–log scale. Reprinted with permission from ref. 71. Copyright 2007 American Physical Society. (**e**) Third harmonic generation as a function of SAM thickness in particle-on-film geometry (inset), comparing the measurements (black line) with the classical (blue line) and QCM (red line) calculations. Insets show near-field distributions simulated using QCM and local Drude models. Reprinted with permission from ref. 73. Copyright 2014 American Chemical Society.

One technique to probe plasmonic field enhancement is surface-enhanced Raman scattering^14^,^15^, in which vibrational transitions in molecules are probed indirectly using inelastic Raman scattering of visible or near infrared light. The cross-section of this process scales approximately as the fourth power of the near-field enhancement. Zhu *et al*.^70^ measured the SERS enhancements of thiophenol molecules adsorbed on nanoparticle dimers as a function of gap distance (Fig. 5a). Quenching of the SERS enhancement was observed below the threshold tunnel-distance that was identified through far-field measurements. QCM calculations that were performed to predict the SERS signals (that is, near-field) and plasmonic spectra (that is, far-field) were found to be consistent with the experimental results, providing support for attributing the quenching of the plasmonic enhancement to electron tunnelling.

Photoluminescence (PL) from plasmonic structures also depends on local plasmonic field enhancements. Kravtsov *et al*.^72^ measured PL from a system consisting of a sharp AFM tip positioned over an atomically flat gold surface (Fig. 5b). The maximum PL intensity was achieved for a gap distance *d*≈1.5 nm, with quenching occurring for smaller gap widths (Fig. 5c). This was accompanied by blue-shifting of the PL spectral peaks for smaller gaps—an indication of the onset of electron tunnelling.

Nonlinear optical processes can also be boosted by plasmonic enhancements. In several studies, a change in the trend of nonlinear signals was reported for subnanometre gaps. For example, Danckwerts *et al*.^71^ observed a slower increase than expected of the enhancement for four-wave mixing in subnanometre gaps in an STM configuration. The four-wave mixing count rate was observed to vary with separation distance *d*, as ∝*d*^−1.8^ for large gaps, but evolved into a weaker dependence (∝*d*^−0.5^) for gaps narrower than *d*≈2 Å (Fig. 5d). Recently, Hajisalem *et al*.^73^ studied third harmonic generation in a particle-on-film geometry where the subnanometre gap was formed via molecular spacers. The third harmonic intensity dropped as the gap distance decreased to 5.1 Å (Fig. 5e), again in agreement with the QCM simulations.

## Photo-induced current in plasmonic gaps

As has been addressed both theoretically and experimentally, the coupling of light and electronic excitations leads to measurable modifications of the electron transport across a junction^104^,^105^. Among the processes that trigger photocurrent in nanogaps, optical rectification^57^ is a second-order nonlinear process that enables simultaneous access to tunnelling currents and their corresponding plasmonic fields. We focus this section on the aspects of optical rectification connected with quantum effects in plasmonic gaps. Additional information about other aspects involving thermal and band structure effects can be found in a recent work by Stolz *et al*.^106^

**Figure 6:**
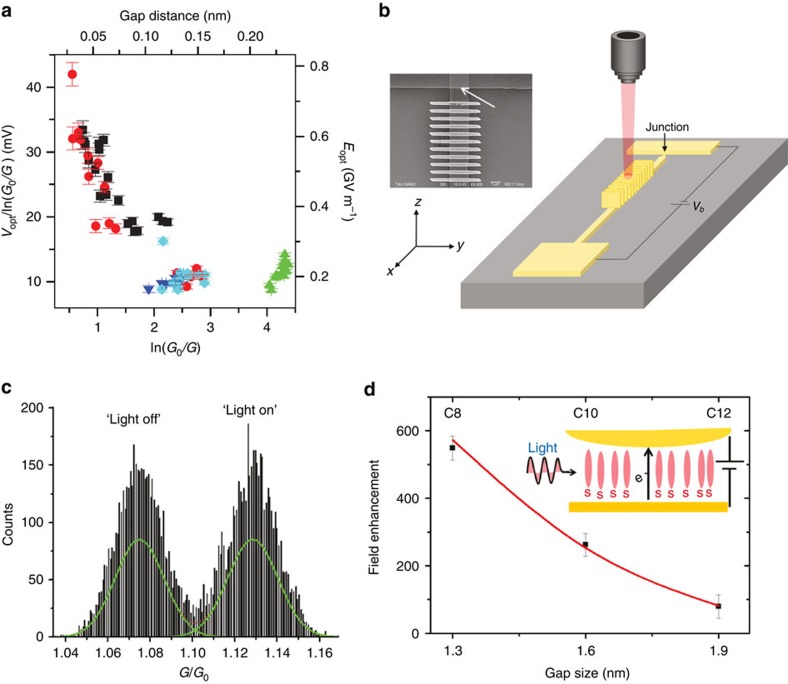
Photo-induced current in plasmonic gaps. (**a**) Optical rectification voltage *V*_opt_ as a function of gap distance (top axis) or junction conductance *G* (bottom axis) for nanogap junctions fabricated via electromigration. Error bars indicate the statistical uncertainty in *V*_opt_ and ln *G*. Image reproduced from ref. 93. Copyright 2010 Nature Publishing Group. (**b**) Schematic illustration of an Au electrical plasmon detector. Creation of SPPs is achieved by normal illumination of the grating by a laser via a microscope objective. (**c**) SPP-modulated conductance histogram measured with the set-up in **b**. Irradiation appears to shift the Gaussian describing the distribution of conductance values around ∼1*G*_0_ (‘light-off' conductance) to a new and higher mean value (‘light-on' conductance). (**b**,**c**) Images are reprinted with permission from ref. 107. Copyright 2011 American Chemical Society. (**d**) Field enhancement in a plasmonic nanogap estimated from the rectification current. Reprinted with permission from ref. 108. Copyright 2011 American Chemical Society.

Ward *et al*.^93^ first demonstrated that a *dc* rectification current can be generated in subnanometre gaps upon optical illumination. This current is generated by nonlinear electron tunnelling assisted by plasmonic resonances, making it (and therefore the voltage, Fig. 6a) closely related to the gap distance. In another work, Ittah *et al*.^107^ studied plasmon-modulated current across quantum contacts made of a few gold atoms. A grating formed at one of the electrodes allows coupling of the incident light to propagating plasmons, which are launched towards the gap junction (Fig. 6b). The change of conductance as a result of the incident light—and thus the launching of plasmons—is shown in Fig. 6c, but is not easy to directly disentangle from induced thermal effects.

Plasmon enhanced rectification of the tunnelling current and the associated increase of conductance has been also reported in molecular junctions^108^,^109^. For instance, in a junction filled with thiol-based self-assembled monolayers, the number of carbon atoms in the molecules determines the gap distance^108^. The magnitude of the rectified current can thus be used to estimate the field enhancement as a function of the gap distance. For a gap distance of 1.3 nm, the enhancement was estimated to be as large as 550-fold (Fig. 6d). However, the molecular conductance in this situation was insufficient to trigger quenching of the plasmonic near-field.

## Molecular control over quantum plasmonic systems

**Figure 7:**
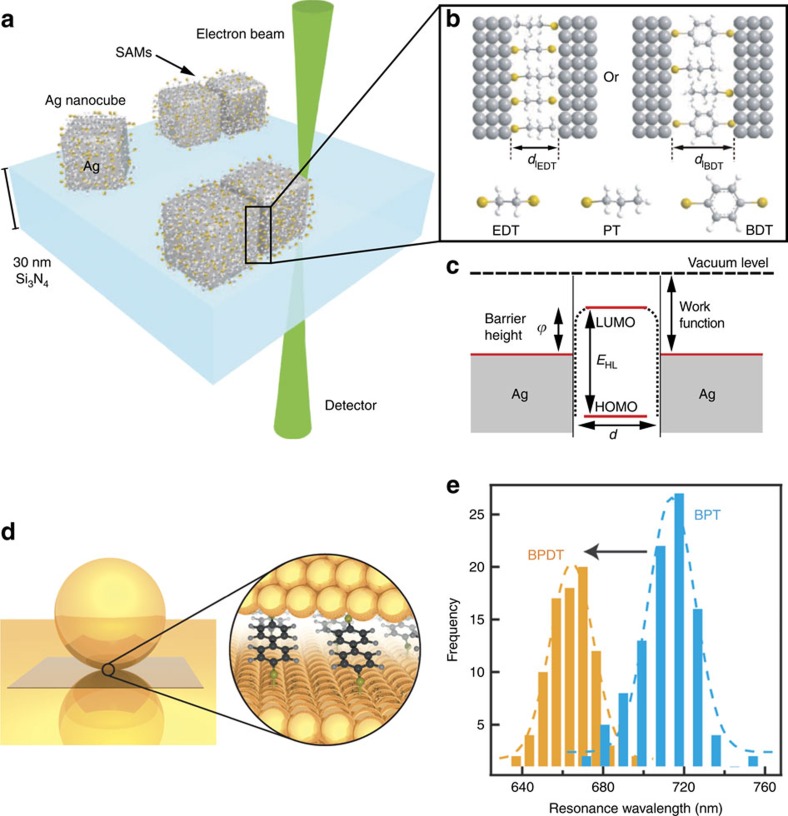
Plasmonic tuning using molecular junctions. (**a**) Schematic illustration of a molecular junction made of two Ag nanoparticles linked by a SAM layer. (**b**) The distance between the two nanoparticles is determined by the thickness of the SAMs. (**c**) A charge transfer channel between the Ag nanoparticles can be opened by lowering the tunnelling barrier using molecules. (**a**–**c**) Images reproduced from ref. 97. Copyright 2014 AAAS. (**d**) Schematic illustration of the molecular junction formed in particle-on-film geometry. (**e**) Plasmon resonance wavelengths can be tuned by controlling the concentration of the two types of molecules (biphenyl-4-thiol (BPT) and biphenyl-4,4'-dithiol (BPDT)). (**d**,**e**) Images are reprinted with permission from ref. 112. Copyright 2015 American Chemical Society.

If well controlled, the spectral changes associated with quantum effects in plasmonic gaps could lead to new applications in the fields of optical spectroscopy^83^ and optoelectronics^110^,^111^. Recently, Tan *et al*.^97^ showed that plasmon resonances can be tuned via molecular tunnel junctions. The junctions comprised two Ag nanocubes on whose surfaces self-assembled monolayers of thiol-group molecules had been attached (Figs 3d and 7a). If no molecules were present in the gap, the tunnelling barrier would only depend on the work function of the electrode material and the gap distance. The presence of the molecules, however, lowers the tunnelling barrier by an amount that depends on the electronic structure of the molecules (Fig. 7b,c). For example, electron tunnelling across a vacuum junction with gap distance of 1.3 nm is negligible. However, as the gap is bridged by the molecules, CTP modes are observed at this separation, indicating the presence of through-molecule tunnelling. This makes it possible to switch the CTP modes by controlling the properties of the molecules in the gap.

In a similar application, Benz *et al*.^112^ demonstrated plasmonic tuning in the nanoparticle-on-mirror configuration by using two groups of molecules as the spacer (Fig. 7d). The biphenyl-4,4'-dithiol (BPDT) molecules have two thiol groups that bond to Au surfaces, permitting conductive links across the gap to be formed. The biphenyl-4-thiol (BPT) molecules lack the second thiol group and are therefore not able to create a conductive link. It was shown that a spacer comprising both types of molecules could be formed. By adjusting the relative concentrations of the molecules, the conductivity of the junction could be tuned. A large tuning range (up to 50 nm) for the peak wavelength of the plasmonic resonance was demonstrated (Fig. 7e). Benz *et al*. also noted that this could enable quantification of the number of molecules in a nanoscale gap, provided that the conductance of the individual molecule was known. Similar tunability of the plasmonic response has also been reported for a sphere dimer forming a junction functionalized with alkanedithiols^113^,^114^, whose change in length is associated with a modification of the molecular conductance.

A number of other exciting prospects could arise from molecular tunnel junctions. One could employ optically switchable molecules in the gap, enabling real-time control over the plasmon resonance, although steric limitations on molecular re-orientation do not make this simple to achieve. One might be able to observe a few molecules on a nanoparticle undergoing chemical reactions. Dark-field optical microscopy over a large field-of-view could enable many such events to be monitored simultaneously. We emphasize that the molecular tunnel junction is a workhorse of this field, but normally only characterized with *dc* or low-frequency *ac* measurements. In the two studies described in this section, molecular conductances are probed at optical frequencies. This could open up a rich area of exploration, due to emergence of phenomena such as electron–electron and electron–vibrational scattering^115^. Indeed, conductive molecules bridging plasmonic junctions have been addressed not only in the context of transport properties^115^,^116^, but also in Raman scattering studies^117^,^118^,^119^,^120^.

## Perspectives and future directions

Recent experimental advances have enabled fabrication of metallic structures with subnanometre gaps, thereby bringing plasmonics into the regime of quantum mechanics. Nonlocal screening and the coupling between tunnelling electrons and photons enhanced by the strong electromagnetic fields in a plasmonic gap lead to new phenomena that are of fundamental and practical interest, and furthermore challenge existing theories. Fully quantum-mechanical simulations of large plasmonic structures are still not practical and will remain challenging in the near future. Several semi-classical approaches have been proposed that account for quantum effects within classical electromagnetic theory. The predictions of these models regarding the interplay between photons and electron dynamics at optical frequencies have been backed up both by far- and near-field experimental observations, although in some cases only qualitatively. Despite careful studies correlating plasmonic resonances and physical gap distances, knowing the precise morphology of the gap region would greatly facilitate progress in this field. Recent advances in electron tomography could be very fruitful for this^121^. In addition, theoretical studies have predicted that the optical response is sensitive to changes in the conductance that arise from atomic-scale rearrangements in the junction^122^,^123^. The ability to precisely reconstruct the gap morphology would allow these phenomena to be studied experimentally. A result of such studies might be the emergence of optics as a probe for atomic-scale electron transport.

The link between plasmon response and electron transport through gap junctions is of fundamental interest as it represents the physical mechanism that governs how the plasmonic response behaves in the transition from separated to overlapped nano-objects. In particular, by properly accounting for this effect, the divergence in the response predicted by the classical approach is corrected for. In addition to the use of plasmonics for field-enhanced spectroscopies and nonlinear effects, the opportunity to exploit quantum effects in the interplay between optics and electron transport in practical applications is one of the most exciting prospects of the field. Indeed, it was recently pointed out that by controlling electron transport one might tune the plasmon response of the nanostructure^112^. STM^124^ and molecular electronics^97^ present opportunities to achieve active control through narrow junctions in a flexible manner as the gap distance can be readily adjusted. Other methods for achieving this control might include applying a *dc* bias across a junction^125^,^126^ or introducing a gate electrode in a junction functionalized with self-organized molecular layers^127^. This might enable fast and versatile electrical control over the plasmon response of metallic nanostructures in the optical range, similar to that widely reported for graphene structures at lower frequencies^128^. The role that such control would play in the tuning of plasmon-exciton couplings should not be underestimated. Conversely, the coupling between tunnelling electrons and photons leads to plasmon generation and photon emission from inelastic tunnelling events^129^, thereby realizing nanoscale localized light sources^124^,^130^,^131^. These represent a new paradigm in optoelectronics, motivating experimental and theoretical efforts aimed at understanding the underlying physics.

Although the studies performed thus far have been mainly concerned with materials such as gold and silver, quantum mechanical effects should also be considered for other plasmonic materials^7^, such as aluminum, conductive oxides and nitrides, as well as two-dimensional materials such as graphene. We also note that the possibilities for applications of molecular tunnel junctions go beyond the simple option of plasmonic tuning, as the tight confinement of light across the gap as well as the tunnelling electrons could also enable possibilities for controlling the physical and chemical properties of the molecules themselves^115^. With so many directions open for exploration, we foresee that the study of quantum effects associated with subnanometre gaps will continue to be an active research topic as a sub-field of quantum plasmonics^132^.

## Additional information

**How to cite this article:** Zhu, W. *et al*. Quantum mechanical effects in plasmonic structures with subnanometre gaps. *Nat. Commun.* 7:11495 doi: 10.1038/ncomms11495 (2016).

## Figures and Tables

**Figure 1:**
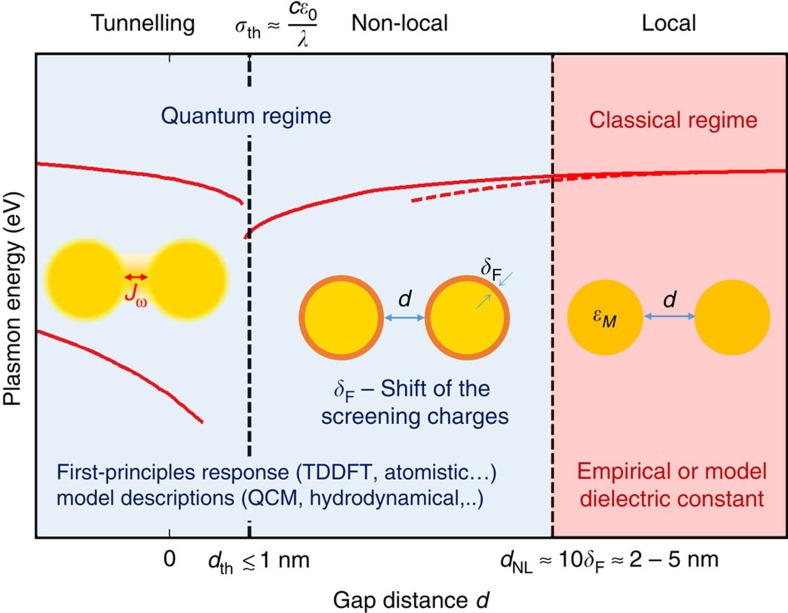
The impact of quantum mechanical effects on plasmonic resonances. Sketch of the different regimes for the plasmon resonances of a sphere-dimer in vacuum identified as a function of the gap distance *d*. These regimes are illustrated by the energies of the plasmonic modes predicted by the classical (red dashed line) and quantum calculations (solid lines). For large gap distances, the system is in the classical regime and its response can be described using Maxwell's equations with empirical or model local dielectric constants of the metal *ɛ*_M_. In the nonlocal regime (*d*<*d*_NL_), the actual position of the screening charges with respect to the geometrical boundaries given by *δ*_F_ leads to an effective correction of the ‘physical' gap distance compared with the geometrical gap distance *d*. The nonlocal screening leads to deviations between the classical (dashed line) and quantum descriptions (solid line). In the tunnelling regime, the *ac* tunnelling current *J*_*ω*_ across the junction strongly changes the optical response, when the conductivity of the junction becomes larger than *σ*_th_ (which sets the corresponding threshold gap distance *d*_th_). The plasmon modes of the separated dimer are progressively extinguished, and the CTP modes emerge before their direct geometrical overlap. We denote the distance range where the electric tunnelling and/or the nonlocal screening are important as the ‘quantum regime'. The plasmonic resonance in this regime can be addressed using *ab-initio* approaches or model descriptions. The transition between the different regimes is smooth and the boundaries shown in the figure are only indicative.

## Semi-classical models of quantum effects in plasmonic structures.

Quantum-corrected model (QCM)

The computational limitation of time-dependent density functional theory (TDDFT) simulations has motivated the development of semi-classical models. In the QCM^64^, the plasmonic response is determined by solving Maxwell's equations, but with electron tunnelling accounted for by ascribing an effective dielectric function *ɛ*_g_(*l*,*ω*) to the gap (schematic **a** in the figure of the box). Here, *l* is the ‘local' separation distance (schematic **b** in the figure of the box) that varies at different gap positions, and *ω* is the optical frequency. This function is parameterized in a manner that leads to its corresponding conductivity

where *ɛ*_0_ is the vacuum permittivity, being equal to the *dc* tunnelling conductivity *σ*_0_ in the static limit (*ω*=0), as determined from a quantum mechanical calculation of the tunnelling probability *T*(*l*) across the potential barrier (schematic **b** in the figure of the box). This leads to the relationship between the field and the tunnelling current across the gap being correctly reproduced. More specifically, a convenient approach to parameterize *ɛ*_g_(*l*,*ω*), keeping the model simple and facilitating the extrapolation of the gap dielectric function to the situation of full contact (*l*=0), is to consider a Drude-type permittivity:

where *ɛ*_∞_(*l*,*ω*) is a background dielectric function that depends on the particular nature of the metal and on the surrounding material (*ɛ*_∞_(*l*,*ω*)=1 for a pure Drude-like metal in vacuum), *ω*_g_ is the plasma frequency of the metal that constitutes the plasmonic structure and *γ*_g_(*l*) is the effective damping term. The latter is taken as having an exponential dependence on the distance *l*, as this is typical for tunnelling processes:

Here *γ*_g_(*l*=0) is the damping frequency of the metal and *q* is a material-dependent characteristic parameter that is retrieved by requiring that *σ*_g_(*l*,*ω*=0)=*σ*_0_ for a given distance as discussed above. Here, we have presented a Drude-type approach to construct the QCM. However, if computationally feasible, *σ*_g_(*l*,*ω*) could be obtained directly from fully time-dependent quantum mechanical calculations^85^, and equation (1) could be inverted to obtain the *ɛ*_g_(*l*,*ω*) needed for the QCM. The major advantage of the QCM is that it is practical for use with large plasmonic structures, as standard solvers of classical electromagnetic equations can be employed with electron tunnelling taken into account.

**Pictorial description of the QCM.** (**a**) Schematic of a plasmonic gap where the gap is described by means of an effective local gap dielectric function *ɛ*_g_(*l*,*ω*) which depends on the gap separation, *l*, at each point. (**b**) Model configuration of a flat metal-insulator-metal cavity of width l that allows determination of the electron transmission probability *T(l)*, used as an input in *ɛ*_g_(*l*,*ω*).

Nonlocal hydrodynamic model (NLHD)

One method for addressing the nonlocal screening consists of using the hydrodynamic model for the motion of electrons in a metal^28^,^29^. In the NLHD, the linearized Navier–Stokes equation is used to describe the motion of the interacting electron gas. The electron current density **J** inside a metal induced by an electric field **E** oscillating at frequency *ω* is governed by:

where *σ*_D_ is the Drude conductivity and parameter *β* is proportional to the Fermi velocity of the electrons. This results in a longitudinal component of the permittivity tensor of the metal in wavevector-space (**k-**space), given by:

The transverse component is still given by the Drude model (equation (5) with *β*=0). This new permittivity tensor of the metal is then used when solving the Maxwell equations. It can be seen why this dielectric function corresponds to a ‘nonlocal' model—its dependence on propagation vector means that the electric displacement **D** does not depend solely on the electric field **E** at a particular location, but also on **E** in the surrounding medium.
